# Survival analysis of patients with hepatocellular carcinoma based on the ratio of platelet count to spleen diameter

**DOI:** 10.3389/fphar.2024.1449603

**Published:** 2025-01-06

**Authors:** Huiwen Yan, Dongdong Zhou, Xiaoli Liu, Peng Wang, Tingting Jiang, Zhiyun Yang

**Affiliations:** ^1^ Center for Integrative Medicine, Beijing Ditan Hospital, Capital Medical University, Beijing, China; ^2^ Rehabilitation Medicine, People's Hospital of Daxing'anling Region, Daxing'anling, Heilongjiang, China

**Keywords:** hepatocellular carcinoma, the ratio of platelet count to spleen diameter, LASSO Cox regression, nomogram, prognosis

## Abstract

**Background:**

In China, 80% of Hepatocellular Carcinoma (HCC) is associated with cirrhosis. Portal hypertension, the most common outcome of cirrhosis progression, has a high incidence. Platelet count/spleen diameter ratio (PSL) with a cut-off value of 909 can predict the presence of esophagogastric varices and thus portal hypertension, which is also an independent risk factor for early recurrence and late recurrence of hepatocellular carcinoma after resection. Therefore, the effect of PSL on the overall survival (OS) of patients with HCC is necessary. The aim of this study was to apply a new method to establish and validate a model for predicting the prognosis of patients based on PSL with HCC.

**Methods:**

A total of 1,104 patients with clinical diagnosed with HCC following non-surgical therapy randomly divided the patients into a primary cohort and a validation cohort in a ratio of 7:3, in which 772 HCC patients were in the primary cohort and a total of 332 HCC patients were in the validation cohort. Through Lasso-Cox analysis, the independent predictors of OS of training cohort were included in nomogram1, and the independent predictors of Cox regression analysis were included in nomogram2. Nomogram1 and nomogram2 used consistency index (C-index), AUC and time-dependent ROC curves in the training cohort, respectively, and the calibration curves were plotted. All suggest that nomogram1 is better than nomogram2. We get similar results in the validation cohort.

**Results:**

The C-index of nomogram1was 0.792 (95%CI: 0.772–0.812), which was superior to nomogram2 (0.788) and traditional modes (0.631–0.712). The AUC of nomogram1 was 0.866 (95%CI: 0.840–0.889). In the validation cohort, the nomogram1 still gave good discrimination (C-index: 0.769, 95%CI: 0.740–0.798; AUC: 0.867, 95%CI: 0.826–0.902). Calibration plots for 3-year OS probabilities showed the good agreement between nomogram1 predictions and actual observations. In addition, we found that the decision curve analysis of nomogram1 and nomogram2 was also meaningful.

**Conclusion:**

Novel nomogram containing PSL, based on LASSO Cox regression, had higher predictive efficacy for 3-year overall survival in patients with HCC.

## Background

According to the latest global cancer statistics for 2021, Hepatocellular Carcinoma (HCC) is the fourth leading cause of cancer death worldwide (Sung et al., 2021). There are about 906,000 new cases of HCC and 803,000 deaths each year. Among them, the death rate of HCC patients in China accounts for half of the global total (Torre et al., 2015). Therefore, HCC is a major public health problem in China. Platelet count is an independent risk factor for the development of HCC, which may be involved in the development (Akuta et al., 2012; Wang et al., 2013), progression and metastasis of HCC (Franco et al., 2015; Menter et al., 2014). However, the relationship between PLT and HCC prognosis remains controversial. Some studies have found that patients with low PLT have poor prognosis after HCC. (Wu et al., 2012; Roayaie et al., 2013). At the same time, studies have found that HCC patients with high PLT have a high risk of tumor metastasis and vascular invasion and are associated with poor prognosis (Morimoto et al., 2014; Lee et al., 2015). However, the etiology may affect platelets, such as long-term drinking can inhibit bone marrow tissue hyperplasia and hepatitis B virus can affect platelets, so this single index is not stable. In recent years, it has been proposed that the ratio of platelet count (PC) to spleen diameter (SD) (PSL) is better than a single index. Taking 909 as the cutoff value, the presence of esophagogastric varices can be accurately predicted, with a negative predictive value (NPV) of 100%, and the degree of varices can also be evaluated (Colli et al., 2017). Blood routine examination and abdominal B-ultrasound examination are both extremely routine clinical examinations, and the data are easy to obtain, reproducible, and do not increase additional costs, which has obvious cost-benefit advantages (Mangone et al., 2012). Meanwhile, Marasco et al. (2019) showed that PSL < 909 was an independent risk factor for early recurrence and late recurrence after HCC resection, which was related to the prognosis of HCC. However, the study was limited to patients undergoing surgery. Therefore, it is necessary to investigate the effect of PSL on overall survival (OS) of HCC patients.

Predicting the prognosis of HCC patients and grading their risk, and early intervention of patients are effective strategies to improve the survival of HCC patients. The current prognostic models for HCC patients mainly include Okuda, TNM, BCLC, Child and CLIP. The predictors of these prognostic models mainly focused on tumor burden, liver function, life status, etc. At the same time, these classical models were built earlier and contain relatively single predictors, so their predictive effect on some specific groups is unknown (Dongdong et al., 2020). Therefore, it is necessary to use new methods to screen out more comprehensive prognostic predictors and construct simple visual prognostic prediction models.

The least absolute shrinkage and selection operator (LASSO) Cox regression is a method for variable selection and contraction in Cox proportional hazards model proposed by Tibshirani (1997) in 1997. LASSO Cox regression analysis was used to construct the penalty function and obtain a more refined model. Our team applied Lasso Cox regression to screen model factors for patients with A-fetoprotein-negative liver cancer, and the prediction effect of the constructed model was significantly higher than other prognostic prediction models (Zhou et al., 2021). However, the use of LASSO Cox regression model of liver cancer patients to screen factors, and then build models, its predictive effect due to other prognostic models, to be further verified.

The aim of this study was to analyze the relationship between platelet count to spleen diameter ratio and prognosis of patients with liver cancer, and to construct a model including platelet count to spleen diameter ratio to predict long-term survival of patients with liver cancer using LASSO Cox regression. In addition, the constructed model was compared with the traditional staging system to determine whether it could provide more accurate prognostic prediction. According to the constructed LASSO Cox regression model, different prognostic risk groups were identified, and early intervention was carried out to reduce the mortality of patients.

## Methods

### Patient selection

A retrospective study was conducted on 2,580 patients with HCC treated at Beijing Ditan Hospital of Capital Medical University from January 2012 to December 2017. The Ethics Committee of Beijing Ditan Hospital approved the study and waived the requirement for informed consent. Patients with HCC were initially included based on the results of ultrasound, computed tomography (CT), and magnetic resonance imaging (MRI). The exclusion criteria were: (1) human immunodeficiency virus (HIV) infection; (2) metastatic liver cancer; (3) incomplete data; and (4) having undergone radical surgical treatments.

Patients who underwent radical surgical treatments were excluded to focus on the prognostic assessment of those receiving non-surgical therapies. This focus allows for the development of a prognostic model tailored to patients who are not candidates for surgery, which is particularly important since a substantial proportion of HCC patients present at an advanced stage or have comorbidities precluding surgical intervention.

The definition of comprehensive treatment with traditional Chinese medicine (TCM) was the documented use of TCM in medical records after the diagnosis of HCC. Finally, 1,104 HCC patients with complete clinical data were randomly divided into a training cohort and a validation cohort in a ratio of 7:3. The follow-up period for this study was 3 years or until the patient’s death, with the final deadline for follow-up set as December 2020.

### Demographics and clinical data

The data provided by patients for the first visit include: general background, such as gender, age, smoking history, drinking history, family history of HBV, family history of HCC, traditional Chinese medicine (TCM), etiology; Laboratory indicators like white blood cell count (WBC), neutrophil to lymphocyte ratio (NLR), red blood cell count (WBC), hemoglobin (HGB), platelet (PLT), platelet count/spleen diameter ratio (PSL), alanine aminotransferase (ALT), aspartate aminotransferase (AST), total bilirubin (TBIL), albumin (ALB), lactate dehydrogenase (LDH), gamma-glutamyl transpeptidase (GGT), alkaline phosphatase (ALP), acetylcholinesterase (CHE), total cholesterol (TC), triglyceride (TG), creatinine (CR), prothrombin time activity (PTA), C reactive protein (CRP); Tumor-related features like alpha fetoprotein (AFP), portal vein tumor thrombus (PVTT).

### Statistical analysis

In this study, we used SPSS 24.0 (IBM, Chicago, IL, USA), R software (version 3.6.3; http://www.Rproject.org) and MedCalc 19.2.0 for statistical analysis. We implemented a stratified random sampling approach to divide patients into training and validation cohorts in a 7:3 ratio, ensuring similar distributions of key clinical variables across both cohorts. The stratification was performed based on the outcome variable to maintain balance in prognostic factors. The randomization process was conducted using the “caret” package. The “glmnet” package in R 3.6.3 is used for Lasso-Cox regression and “nomogramEx” package is used to build nomogram. On the basis of establishing nomogram, “foreign”, “survival”, “nricens”, “stdca” and “rms” packages are used to analyze C index and calibration curve, to score NRI and IDI, and to draw decision curve. Analyze and draw time-dependent ROC curves with the “time ROC” package. The total score of each patient was calculated according to nomogram, and the patients with high, medium and low prognostic risk were divided into three groups according to the quartile. MedCalc 19.2.0 was used to draw Kaplan-Meier curves in low risk group, medium risk group and high risk group. The two-tailed *P*-value < 0.05 was statistically significant.

## Results

### Basic characteristics

A total of 1104 HCC patients who met the inclusion and exclusion criteria were included in this study. The patients were randomly divided into the modeling cohort and the validation cohort at a ratio of 7:3, with 772 HCC patients in the modeling cohort and 332 HCC patients in the validation cohort. To enhance the objectivity and simplicity of the model, continuous variables were converted to categorical variables using clinically normal laboratory test values as cutoffs.

The majority of the study population was male (80.3% vs. 80.1%; *P* = 0.942), patients older than 50 years (77.6% vs. 79.5%; *P* = 0.477). More than half of the patients using TCM (51.4% vs. 54.5%; *P* = 0.444). In addition, the majority of patients had cirrhosis (90.9% vs. 89.5%; *p* = 0.444), with HBV infection as the main cause (95.9% vs. 97.3%; *p* = 0.510), with HBsAg positivity (70.5% vs. 66.6%; *p* = 0.198). More than half of the patients had platelet/spleen diameters <909 (67.9% vs. 79.3%; *p* = 0.646); Almost half of the patients in TNM III (51.0% vs. 50.6%; *P* = 0.661); Most patients were still in stage 0-B BCLC (69.1% vs. 74.4%; *P* = 0.245), child-Pugh A-B patients accounted for the majority (85.6% vs. 88.8%; *P* = 0.238), (73.7% vs. 70.5%; *P* = 0.270) had tumor diameter <5 cm (Table 1).

**TABLE 1:** Characteristics of the clinical profile of the modeling group and validation group of patients with HCC.

Characters	Training cohort (n = 772)	Validation cohort group (n = 332)	*p*-value
General Features			
Gender (Female/Male)	152/620 (19.7/80.3)	66/266 (19.9/80.1)	0.942
Age (<50/≥50)	173/599 (22.4/77.6)	68/264 (20.5/79.5)	0.477
Smoke (No/Yes)	465/307 (60.2/39.8)	201/131 (60.5/39.5)	0.923
Drink (No/Yes)	481/291 (62.3/37.7)	222/110 (66.9/33.1)	0.148
Hypertension (No/Yes)	560/212 (72.5/27.5)	245/87 (73.8/26.2)	0.667
DM (No/Yes)	579/193 (75.0/25.0)	269/63 (81.0/19.0)	0.030
Hyperlipidemia (No/Yes)	726/64 (94.0/6.0)	312/20 (94.0/6.0)	0.966
CHD (No/Yes)	750/22 (97.2/2.8)	324/8 (97.6/2.4)	0.680
Cirrhosis (No/Yes)	70/702 (9.1/90.9)	35/297 (10.5/89.5)	0.444
HS (No/Yes)	510/262 (66.1/33.9)	205/127 (61.7/38.3)	0.169
Family history of HBV (No/Yes)	539/233 (69.8/30.2)	238/94 (71.7/28.3)	0.533
Family history of HCC (No/Yes)	745/27 (96.5/3.5)	324/8 (97.6/2.4)	0.344
TCM (No/Yes)	375/397 (48.6/51.4)	151/181 (45.5/54.5)	0.345
Etiology			
HBV	740 (95.9)	323 (97.3)	0.510
HCV	10 (1.3)	3 (0.9)	
Others	22 (2.8)	6 (1.8)	
Complications			
Ascites (No/Yes)	435/337 (56.3/43.7)	186/146 (56.0/44.0)	0.921
HE (No/Yes)	754/18 (97.7/2.3)	325/7 (97.9/2.1)	0.819
Laboratory indicators			
WBC (≤10/>10*10^9/L)	751/21 (97.3/2.7)	323/9 (97.3/2.7)	0.993
NLR (≤2/>2)	291/481 (37.7/62.3)	136/196 (41.0/59.0)	0.306
RBC (<4/≥4*10^12/L)	387/385 (50.1/49.9)	172/160 (51.8/48.2)	0.609
HGB (<110/≥110 g/L)	194/578 (25.1/74.9)	75/257 (22.6/77.4)	0.367
PLT (<100/≥100*10^9/L)	472/300 (61.1/38.9)	209/123 (63.0/37.0)	0.570
PSL (<909/≥909)	524/248 (67.9/32.1)	230/102 (69.3/30.7)	0.646
ALT (≤50/>50 U/L)	576/196 (74.6/25.4)	255/77 (76.8/23.2)	0.438
AST (≤40/>40 U/L)	405/367 (52.5/47.5)	186/146 (56.0/44.0)	0.276
TBIL (≤18.8/>18.8 μmol/L)	389/383 (50.4/49.6)	160/172 (48.2/51.8)	0.503
ALB (<40/≥40 g/L)	534/238 (69.2/30.8)	234/98 (70.5/29.5)	0.664
LDH (≤250/>250 U/L)	673/99 (87.2/12.8)	295/37 (88.9/11.1)	0.436
GGT (≤60/>60 U/L)	426/346 (55.2/44.8)	188/144 (56.6/43.4)	0.658
ALP (≤125/>125 U/L)	560/212 (72.5/27.5)	248/84 (74.7/25.3)	0.458
CHE (≤4,000/>4000 U/L)	345/427 (44.7/55.3)	144/188 (43.4/56.6)	0.687
TC (≤18.8/>18.8 mmol/L)	753/19 (97.5/2.5)	327/5 (98.5/1.5)	0.318
TG (≤1.71/>1.71 mmol/L)	728/44 (94.3/5.7)	310/22 (93.4/6.6)	0.551
CR (≤111/>111μmoI/L)	737/35 (95.5/4.5)	313/19 (94.3/5.7)	0.401
PTA (<70/≥70%)	275/497 (35.6/64.4)	115/217 (34.6/65.4)	0.754
AFP (≤400/>400 ng/mL)	521/251 (67.5/32.5)	230/102 (69.3/30.7)	0.559
CRP (<5/≥5 mg/L)	209/563 (27.1/72.9)	98/234 (29.5/70.5)	0.406
Child			
A	403 (52.2)	171 (51.5)	0.238
B	258 (33.4)	124 (37.3)	
C	111 (14.4)	37 (11.2)	
HBV-related features			
HBV-DNA (≤500/>500 IU/mL)	330/442 (42.7/57.3)	156/176 (47.0/53.0)	0.193
HbsAg (≤250/>250 IU/mL)	228/544 (29.5/70.5)	111/221 (33.4/66.6)	0.198
HBeAg (No/Yes)	453/319 (58.7/41.3)	197/135 (59.3/40.7)	0.838
Tumor-related features			
Tumor multiplicity (Single/Multiple)	434/338 (56.2/43.8)	172/160 (51.8/48.2)	0.177
Tumor size (<5 cm, ≥5 cm)	569/203 (73.7/26.3)	234/98 (70.5/29.5)	0.270
PVTT (No/Yes)	451/321 (58.4/41.6)	202/130 (60.8/39.2)	0.452
BCLC			
0	42 (5.4)	16 (4.8)	0.245
A	240 (31.1)	101 (30.4)	
B	252 (32.6)	130 (39.2)	
C	127 (16.5)	48 (14.5)	
D	111 (14.4)	37 (11.1)	
TNM			
I	229 (29.7)	98 (29.5)	0.661
II	138 (17.9)	64 (19.3)	
III	394 (51.0)	168 (50.6)	
IV	11 (1.4)	2 (0.6)	

### Biomarker selection

**FIGURE 1:**
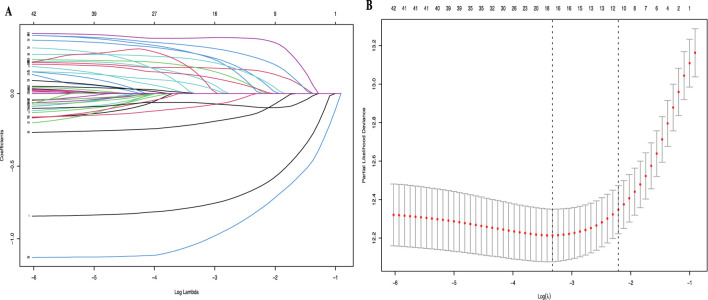
**(A)** LASSO coefficient profiles of the 42 risk factors; **(B)** 11 risk factors selected using LASSO Cox regression analysis. The two dotted vertical lines were drawn at the optimal scores by minimum criteria and 1-s.e. criteria.

All available clinical indicators listed in Table 1, including general patient characteristics, etiology, comorbidities, laboratory indicators, hepatitis B-related characteristics and tumor-related characteristics, were analyzed using the LASSO Cox regression model to identify potential prognostic factors. All available clinical indicators were subjected to LASSO Cox regression, with a significant correlation between TCM, ascites, PVTT, tumor number, tumor size, RBC, HGB, PSL, CR, TBIL, LDH, GGT, ALP, CHE, TC, AFP and OS at minimum values (Figure1A); Further disciplinary regression was performed to take 1-s.e. criteria TCM, ascites, PVTT, tumor number, tumor size, RBC, PSL, TBIL, GGT, ALP and CHE as independent risk factors for HCC (Figure 1B).

Cox univariate analysis of all clinical indicators revealed significant associations between OS and factors such as TCM usage, cirrhosis, HS, ascites, HE, PVTT, HBV-DNA, tumor number, tumor size, NLR, RBC, HGB, PSL, CR, AST, TBIL, ALB, LDH, GGT, ALP, CHE, PTA, AFP and CRP. Subsequent Cox multivariate analysis identified TCM usage, PVTT, tumor number, tumor size, PSL, CR, LDH, GGT and ALP were independent risk factors for prognosis in HCC (Table 2). And the COX analysis results of PFS were shown in Supplementary Table S1.

**TABLE 2:** Univariate and multivariate cox hazards analysis of the training cohort with OS.

	Univariate analysis		Multivariate analysis	
Characteristic	HR (95% CI)	*p*	HR (95% CI)	*p*
TCM				
No/Yes	0.319 (0.265–0.385)	<0.001	0.423 (0.346–0.518)	<0.001
Cirrhosis				
No/Yes	2.655 (1.761–4.004)	<0.001		
HS				
No/Yes	2.369 (1.978–2.838)	<0.001		
PHT				
No/Yes	2.206 (1.833–2.655)	<0.001		
Ascites				
No/Yes	2.772 (2.311–3.325)	<0.001		
HE				
No/Yes	2.013 (1.223–3.315)	0.006		
PVTT				
No/Yes	2.621 (2.189–3.139)	<0.001	1.482 (1.215–1.808)	<0.001
HBV-DNA (IU/mL)				
≤500, >500	1.295 (1.080–1.552)	0.005		
Tumor multiplicity				
Single/Multiple	1.771 (1.481–2.117)	<0.001	1.308 (1.084–1.579)	0.005
Tumor size (cm)				
<5, ≥5	1.912 (1.576–2.320)	<0.001	1.442 (1.153–1.803)	0.001
NLR				
≤2, >2	1.641 (1.355–1.988)	<0.001		
RBC (10^12/L)				
<4, ≥4	0.393 (0.326–0.473)	0.001		
HGB (g/L)				
<110, ≥110	0.416 (0.344–0.503)	<0.001		
PLT (*10^9/L)				
<100,≥100	0.353 (0.287–0.435)	<0.001		
PSL				
<909,≥909	0.206 (0.160–0.265)	<0.001	0.317 (0.220–0.458)	<0.001
CR (μmoI/L)				
<111, ≥111	2.542 (1.764–3.663)	<0.001	2.200 (1.254–3.858)	0.006
AST (U/L)				
<40, ≥40	2.083 (1.739–2.494)	<0.001		
TBIL (μmol/L)				
<18.8, ≥18.8	2.473 (2.056–2.975)	<0.001		
ALB (g/L)				
<40, ≥40	0.423 (0.340–0.527)	<0.001		
LDH(U/L)				
<250, ≥250	2.057 (1.621–2.611)	<0.001	1.555 (1.204–2.008)	0.001
GGT (U/L)				
<60, ≥60	1.931 (1.615–2.308)	<0.001	1.336 (1.078–1.656)	0.008
ALP(U/L)				
≤125, >125	3.017 (2.503–3.636)	<0.001	1.481 (1.177–1.862)	0.001
CHE(U/L)				
≤4,000, >4,000	0.347 (0.289–0.417)	<0.001		
PTA (%)				
<70, ≥70	0.413 (0.345–0.495)	<0.001		
AFP (ng/mL)				
≤400, >400	1.372 (1.138–1.654)	0.001		
CRP (mg/L)				
<5, ≥5	1.253 (1.022–1.536)	0.030		

### Establish and evaluate nomogram

**FIGURE 2:**
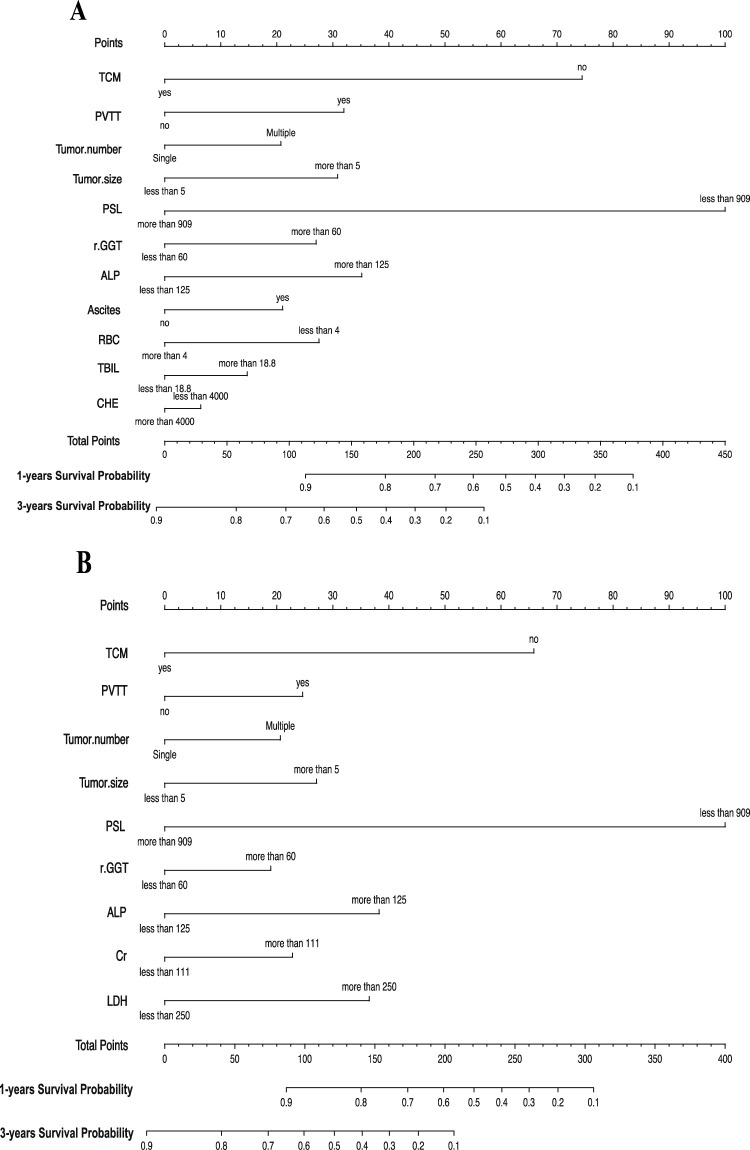
**(A)** Nomogram1 including TCM, PVTT, Tumor number, Tumor size, PSL, GGT, ALP, Ascites, RBC, TBIL and CHE, for one- and 3-years overall survival (OS) in patients with HCC; **(B)** Nomogram2 including TCM, PVTT, Tumor number, Tumor size, PSL, GGT, ALP, Cr and LDH, for one- and 3-years overall survival (OS) in patients with HCC. The nomogram1 and nomogram2 are valued to obtain the probability of one- and 3-years survival by adding up the points identified on the points scale for each variable.

Based on the statistical results, we established two prognostic nomograms—Nomogram 1 and Nomogram 2—to predict the overall survival of HCC patients (Figure 2). Nomogram 1 incorporated the following independent prognostic factors: TCM usage, PVTT, tumor number, tumor size, PSL, Cr, LDH, GGT, and ALP. Each variable was assigned a score based on its relative contribution to the prognosis, allowing for individualized risk prediction by summing the total score. Nomogram 2 included the same variables as Nomogram 1, except that the PSL ratio was replaced by its individual components—platelet count and spleen diameter—as separate variables. This nomogram also assigned scores to each variable to calculate a total prognostic score.

**FIGURE 3:**
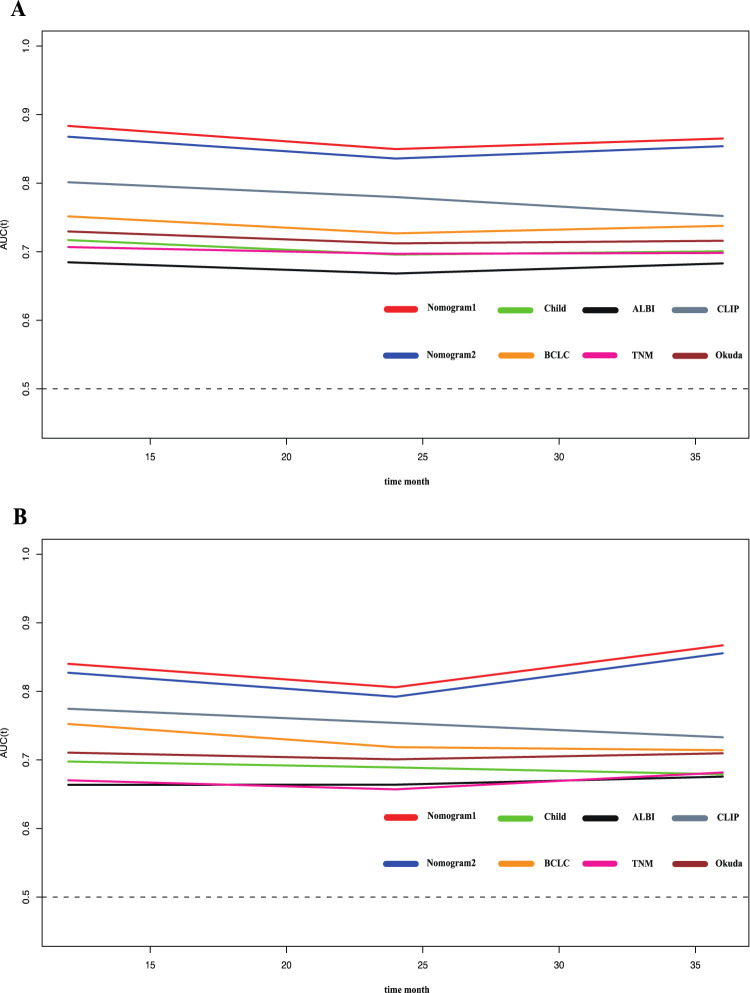
Time-ROC curve of the eight models in the primary and validation cohort. Red line: Nomogram1; Blue line: Nomogram2; Green line: Child-pugh; Yellow line: BCLC stage; Black line: ALBI; Pink line: TNM; Gray line: CLIP; deep red line: Okuda. **(A)** Time-ROC curve of the eight models in the primary. **(B)** Time-ROC curve of the eight models in the validation cohort.

To compare and evaluate the performance of the two nomograms, we calculated the concordance index (C-index) and the area under the receiver operating characteristic curve (AUC). In the modeling cohort, Nomogram 1 had a C-index of 0.792 and an AUC of 0.866, while Nomogram 2 had a C-index of 0.788 and an AUC of 0.854 (Table 3). Time-dependent ROC curves showed that over time, the predictive efficiencies of both models were not significantly different and both significantly outperformed traditional models such as Child-Pugh, BCLC, ALBI, and TNM staging systems (Figure 3).

**TABLE 3:** C-index and AUC of prognostic staging systems for Training and Validation cohort.

	Training cohort				Validation cohort			
Models	C-index	95% CI	AUC	95% CI	C-index	95% CI	AUC	95% CI
Nomogram1	0.792	0.772–0.812	0.866	0.840–0.889	0.769	0.740–0.798	0.867	0.826–0.902
Nomogram2	0.788	0.768–0.808	0.854	0.827–0.878	0.761	0.732–0.790	0.856	0.813–0.892
Child	0.648	0.628–0.668	0.702	0.668–0.734	0.634	0.603–0.665	0.679	0.626–0.729
BCLC	0.676	0.654–0.698	0.736	0.704–0.767	0.665	0.634–0.696	0.714	0.662–0.762
ALBI	0.631	0.609–0.653	0.686	0.652–0.718	0.623	0.590–0.656	0.676	0.622–0.732
TNM	0.648	0.626–0.670	0.697	0.663–0.729	0.625	0.592–0.658	0.682	0.629–0.732
CLIP	0.712	0.690–0.734	0.751	0.719–0.781	0.693	0.660–0.726	0.733	0.682–0.780
Okuda	0.664	0.642–0.686	0.717	0.684–0.749	0.655	0.626–0.684	0.710	0.658–0.758

**FIGURE 4:**
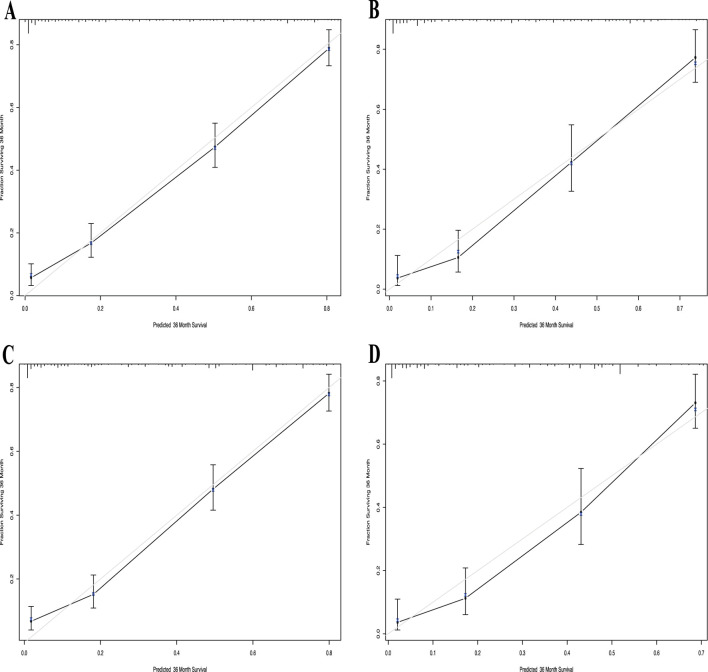
Calibration curve of the nomogram1 and nomogram2 in the primary and validation cohort, with the x-axes are actual survival estimated by the nomogram, the y-axes are observed survival calculated by the Kaplan-Meier method. **(A)** Three-year survival OS in the primary cohort in nomogram1. **(B)** Three-year OS in the validation cohort in nomogram1. **(C)** Three-year survival OS in the primary cohort in nomogram2. **(D)** Three-year OS in the validation cohort in nomogram2.

**FIGURE 5:**
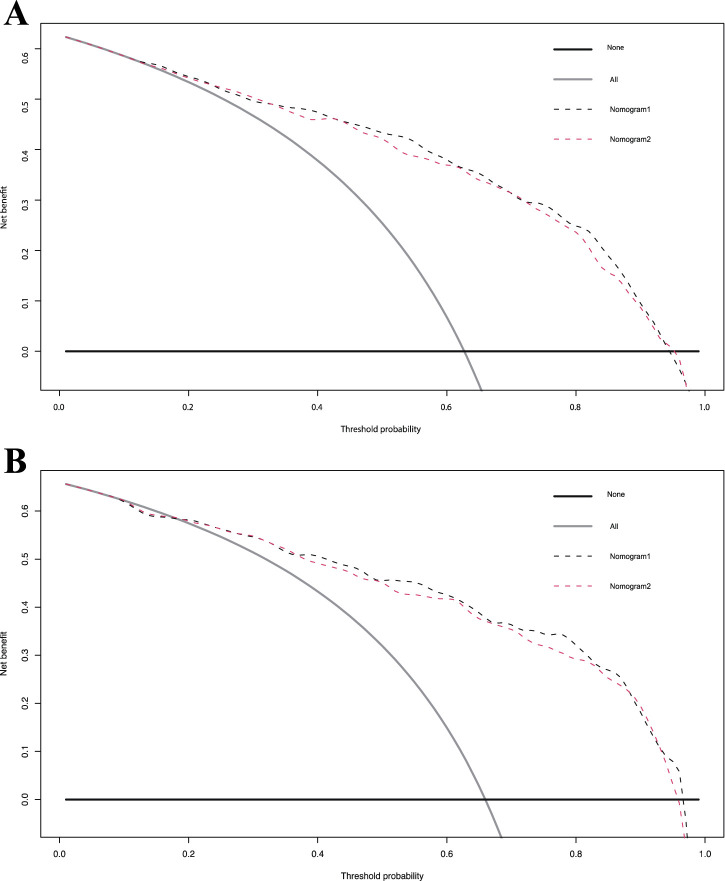
Decision curve analysis for overall survival in the primary and validation cohort. Black line: All patients dead. Gray line: None patients dead. Black dashed line: Model of nomogram1. Gray dashed line: Model of nomogram2. **(A)** Decision curve analysis for overall survival in the primary. **(B)** Decision curve analysis for overall survival in the validation cohort.

The calibration curve for the 3-year OS probability indicated that the predictions made by Nomogram 1 had the best agreement with the actual observations (Figure 4). Decision curve analysis demonstrated that both nomograms provided meaningful clinical net benefits, with Nomogram 1 showing a slightly higher net benefit than Nomogram 2 in the modeling cohort; both yielded a net benefit exceeding 50% (Figure 5). Similar trends were observed in the validation cohort. Therefore, we concluded that Nomogram 1, which incorporates the PSL ratio, is more effective in predicting the prognosis of HCC patients.

### Risk stratification based on nomograms

#### Methods and criteria for risk stratification

To assess the prognostic utility of the nomograms, we divided patients into different risk groups based on their total nomogram scores. Each patient’s total score was calculated using the points specified in the nomogram. Then, based on the quartiles of the total scores, patients were classified into low-risk, middle-risk, and high-risk groups. Specifically, the lowest 25% of total scores constituted the low-risk group, the highest 25% constituted the high-risk group, and the remaining 50% formed the middle-risk group.

#### Survival analyses derived from nomogram 1

**FIGURE 6:**
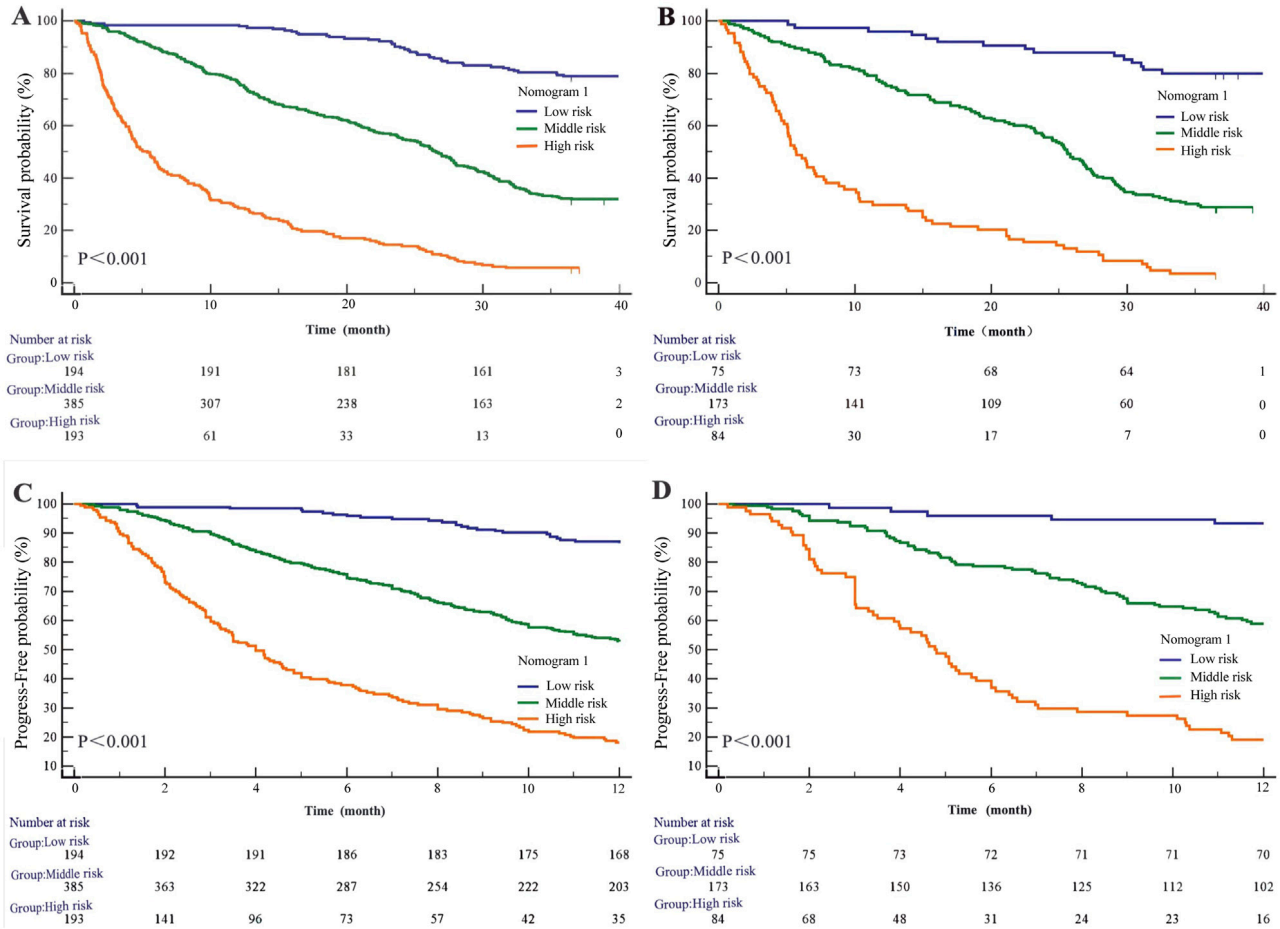
Kaplan-Meier survival curves of nomogram1. **(A)** OS in the primary cohort; **(B)** OS in the validation cohort; **(C)** PFS in the primary cohort; **(D)** PFS in the validation cohort.

Based on Nomogram 1 developed in this study, we stratified patients into low-risk, medium-risk, and high-risk groups, demonstrating good discrimination in both the modeling and validation cohorts. In the modeling cohort (n = 772), the low-, medium-, and high-risk groups comprised 194, 385, and 193 patients, respectively. The median overall survival (OS) was not reached in the low-risk group, while it was 25.57 ± 0.99 months in the medium-risk group and 5.37 ± 0.75 months in the high-risk group (*P* < 0.001). Using the low-risk group as a reference, the hazard ratios (HRs) for OS were 4.46 (95% CI: 3.68–5.41; *P* < 0.001) for the medium-risk group and 14.02 (95% CI: 10.38–18.95; *P* < 0.001) for the high-risk group (Figure 6A). In the validation cohort (n = 332), with 75 low-risk, 173 medium-risk, and 84 high-risk patients, the median OS was not reached in the low-risk group, whereas it was 25.57 ± 0.96 months in the medium-risk group and 5.70 ± 1.11 months in the high-risk group (*P* < 0.001). The HRs for OS were 5.11 (95% CI: 3.82–6.83; *P* < 0.001) for the medium-risk group and 15.08 (95% CI: 9.67–23.51; *P* < 0.001) for the high-risk group compared to the low-risk group (Figure 6B).

Regarding progression-free survival (PFS), in the modeling cohort, the HRs were 4.41 (95% CI: 3.51–5.54; *P* < 0.001) for the medium-risk group and 12.82 (95% CI: 9.37–17.56; *P* < 0.001) for the high-risk group compared to the low-risk group (Figure 6C). In the validation cohort, the HRs for PFS were 6.26 (95% CI: 4.31–9.09; *P* < 0.001) for the medium-risk group and 19.81 (95% CI: 11.99–32.74; *P* < 0.001) for the high-risk group (Figure 6D).

#### Application of the nomogram model

**FIGURE 7:**
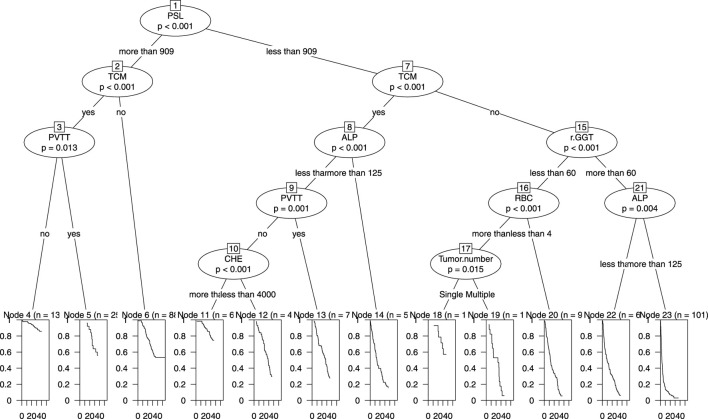
Decision Tree of nomogram1 in the primary cohort including PSL, TCM, PVTT, ALP, GGT, RBC, CHE and tumor number.

We incorporated all the variables from Nomogram 1 into a decision tree in the modeling cohort to visualize the hierarchical importance of prognostic factors (Figure 7). The decision tree revealed that the PSL ratio was the most significant contributor to the model, appearing at the first level. Patients with PSL >909 who were also receiving TCM had significantly higher survival rates compared to those with PSL <909 and not using TCM. This hierarchical stratification underscores the critical role of PSL and TCM usage in influencing patient outcomes.

## Discussion

Patients with hepatitis B virus-associated hepatocellular carcinoma are often associated with liver dysfunction, portal hypertension and splenomegaly, resulting in abnormal platelet counts and abnormal coagulation (Yang et al., 2011). Thrombocytopenia, also known as low platelet count, is common in chronic liver disease due to a lack of sufficient thrombopoietin in normal liver tissue and/or increased platelet destruction due to splenomegaly (Hayashi et al., 2014; Peck-Radosavljevic, 2017). There is much talk about the relationship between PLT counts and HCC prognosis. Roayaie et al. (2013) concluded that a PLT count <150 was associated with a poor prognosis after resection of hepatocellular carcinoma. Morimoto et al. (2014) and Lee et al. (2015) reported that high PLT counts promoted extrahepatic metastasis of HCC and were associated with poor prognosis, respectively. Meanwhile, Kondo et al. found that postoperative survival in HCC patients was not associated with low PLT counts (Kondo et al., 2012). However, their studies did not take into account the fact that platelets may be affected by different factors, such as chronic alcohol consumption, which can inhibit bone marrow tissue proliferation, or hepatitis B virus, which can affect platelet production, suggesting that this single indicator is not stable. Whether the combination of spleen diameter, as a clinically imaging-accessible indicator, would yield a better predictive outcome was the focus of our study.

Various columnar maps have been developed to predict the prognosis of certain cancers and have been shown to be more accurate than traditional staging systems. However, it is mostly limited to forward stepwise Cox regression risk factor screening, which is not conducive to small sample size and multi-indicator model screening (Iasonos et al., 2008). LASSO Cox regression was chosen for this study due to its capacity to handle high-dimensional data and select significant variables in the presence of multicollinearity. By applying a penalty function, this method minimizes overfitting and improves the predictive performance of the model. Compared to traditional stepwise regression, LASSO Cox regression provides a more robust approach for constructing prognostic models in complex clinical datasets (Tibshirani, 1997). A previous study by our team found that model variables for prognosis prediction of AFP-negative HCC patients screened by applying LASSO Cox regression had better precision and resolution than those screened by forward stepwise Cox regression (Zhou et al., 2021).

The study retrospectively included 1104 HCC patients, with the modelling and validation cohorts randomised in a 7:3 ratio. The nomogram1 and nomogram2 were constructed by LASSO Cox regression and Forward Stepwise Cox regression with the selection of independent risk factors. We found that the C index, AUC, time-dependent ROC, specificity, PPV, and calibration curve of nomogram1 were superior to those of nonogram2 in the modelling cohort. They were similar in terms of sensitivity, NPV, and decision curves. Both were significantly higher than traditional models (Child, BCLC, ALBI, TNM, CLIP, Okuda, etc.). We get similar results in the validation cohort. Meanwhile, multiple studies have shown that tumor number, PVTT and tumor size are the main risk factors for predicting OS (Sun et al., 2019; Liu S. et al., 2019; Park et al., 2016; Pang et al., 2015a; Yu et al., 2019; Liu et al., 2020), which is consistent with our findings. The present study found TCM to be an independent risk factor for HCC prognosis, which is the same as our team’s previous findings (Liu X. et al., 2019). Notably, we found that GGT and ALP are key biomarkers closely associated with the progression of HCC disease. Research has shown that preoperative elevation of ALT and GGT is associated with HCC mortality (Xu et al., 2014). The impact of GGT on tumorigenesis may be mediated through intracellular oxidative stress pathways. Numerous pieces of evidence suggest that GGT catalyzed degradation of glutathione can synergistically generate free radicals, leading to lipid peroxidation, which is closely associated with the occurrence of various malignant tumors, including HCC. This may partially explain the association between GGT and HCC (Pompella et al., 2007). An increase in ALP indicates the possibility of biliary obstruction, liver tissue damage, or bone metastasis, further indicating the severity and progression of the disease. Research has shown that ALP, as a marker for the differentiation of embryonic stem cells and other stem cells in bone and adipose tissue, plays a key role in tumor proliferation and progression, and is commonly associated with bile stasis and hepatitis (Yamamoto et al., 2003). Interestingly, the GGT/ALT ratio is a potential effective factor in predicting vascular invasion and prognosis in HBV related HCC patients (Zhao et al., 2021).

However, there are few reports on PSL as a major risk factor for predicting OS in patients. Studies have shown that PLT levels change from initial inflammation through subsequent cirrhosis to the eventual formation of cancer, and that reduced PLT levels increase in-hospital and short-term mortality in patients with liver cancer, in addition to having an impact on long-term survival (Pang et al., 2015b; Maithel et al., 2011). However, the molecular mechanism is not known. In parallel, studies have found that low platelet levels in patients undergoing liver resection and radiofrequency ablation increase the risk of complications and tumour recurrence in patients (Amano et al., 2011; Kaibori et al., 2013). Kubo et al. prospectively included 202 patients with HCV-associated hepatocellular carcinoma undergoing tumour resection and found that only PLT levels were an independent risk factor for prognosis in patients with hepatocellular carcinoma, with levels significantly correlating with the severity of liver fibrosis (*p* < 0.05) (Kubo et al., 2004). It has been shown that a decrease in PLT levels is significantly associated with an increase in AFP levels (Akuta et al., 2012; Kobeisy et al., 2012). PLT levels are usually lower in early tumour stages due to low tumour load, hypersplenism and other factors such as reduced production of thrombopoietin and increased capture of platelets by the spleen (Kondo et al., 2013), where platelets are less affected by tumour factors. In advanced stages of the tumour, the tumour itself secretes more thrombopoietin (Cheng et al., 2019), which increases the platelet count and interacts with cirrhosis and thrombocytopenia. Platelet counts do not fully reflect the extent of cirrhosis. Thus, a single platelet index cannot predict the cirrhosis status of patients and the prognosis of patients with hepatocellular carcinoma. At the same time, higher platelet levels release more proteins, nucleotides and bioactive lipids that promote extravasation of primary liver cancer cells during the interaction of PLT with cancer cells in the bloodstream, sustaining cancer progression (Franco et al., 2015). In assessing the relationship between HCC and PLT, CarRBI et al. observed that PLT stimulated the growth and invasion of HCC cell lines *in vitro* (Carr et al., 2014). Our study also showed that in advanced tumour stages, patients with PVTT but normal or elevated PLT counts had larger tumours than those with thrombocytopenia. This finding also confirms that PLT significantly enhances the high adhesion of hepatocellular carcinoma cell lines. In addition, Tsuguru et al. found that antiplatelet therapy improved overall survival in patients with hepatocellular carcinoma (Hayashi et al., 2020). The spleen also plays a critical role in HCC pathophysiology. Splenomegaly, often associated with portal hypertension in cirrhotic patients, significantly affects platelet kinetics. The enlarged spleen may sequester platelets, leading to thrombocytopenia, which not only reflects liver dysfunction but also alters the systemic inflammatory and immune responses critical to tumor progression. Study shown that preoperative spleen volume is an independent predictor of late recurrence in HCC patients (Yang et al., 2024). By incorporating spleen diameter into PSL, this ratio provides a composite measure that captures both platelet activity and the severity of portal hypertension, making it a potentially robust prognostic indicator for HCC. In this study, PLT was not found to be an independent risk factor for the prognosis of hepatocellular carcinoma. However, in combination with spleen length diameter, the constructed PSL was one of the independent risk factors affecting the prognosis of patients with HCC. This provides new ideas for future research. Furthermore, the application of PSL in different therapeutic contexts warrants exploration. For instance, PSL could be investigated as a biomarker to stratify patients for targeted therapies or immunotherapies. Elevated PSL may indicate patients with lower tumor burden and better immune function, potentially identifying those who are more likely to benefit from immune checkpoint inhibitors. Conversely, patients with reduced PSL may require tailored therapeutic strategies to address underlying portal hypertension and associated complications. These potential applications expand the clinical significance of PSL and warrant further research to validate its utility in guiding personalized treatment strategies.

Despite the strengths of this study, several limitations should be acknowledged. Firstly, as a single-center study, the generalizability of our findings may be limited due to potential data bias. To address this, we recommend future studies to incorporate multicenter cohorts for external validation. Such studies would be critical to confirm the utility of PSL in diverse populations and healthcare settings, ensuring that our prognostic model is broadly applicable in clinical practice. Secondly, we did not explore the mechanisms underlying tumor factor-induced thrombocytopenia in depth, and further research is needed to elucidate these pathophysiological processes. Additionally, one limitation is the absence of detailed data on first-line treatments such as tyrosine kinase inhibitors (TKIs), immune checkpoint blockers (ICBs), chemotherapy, transarterial chemoembolization (TACE), and radiofrequency ablation. Due to the constraints of retrospective data collection, we could not include these variables in our analysis. Future prospective studies should incorporate comprehensive treatment data to validate and refine our prognostic model further, allowing for an assessment of how these therapies interact with the identified prognostic factors.

## Conclusion

In conclusion, this study applied factors from the LASSO Cox regression screening model to develop and validate a visual nomogram for predicting 3-year overall survival in HCC patients based on PSL. In this study, it was shown that PSL <909 is one of the independent risk factors for 3-year overall survival and progression-free survival in HCC patients.

## Data Availability

The raw data supporting the conclusions of this article will be made available by the authors, without undue reservation.
