# Supplementary material for: Survival analysis of patients with hepatocellular carcinoma based on the ratio of platelet count to spleen diameter
Source: Front Pharmacol. 2025 Jan 6;15:1449603. doi: 10.3389/fphar.2024.1449603 (PMC11744000; doi:10.3389/fphar.2024.1449603)
Supplement: Supplementary file 1 [file Table1.docx]

| **Table S1 Univariate and multivariate cox hazards analysis of the training cohort with PFS** | | | | |
| --- | --- | --- | --- | --- |
|  | Univariate analysis |  | Multivariate analysis |  |
| Characteristic | HR (95% CI） | P | HR (95% CI） | P |
| TCM |  |  |  |  |
| (No/Yes) | 0.367(0.306-0.439) | ＜0.001 | 0.565 (0.465-0.687) | ＜0.001 |
| Drink |  |  |  |  |
| (No/Yes) | 1.293(1.085-1.543) | ＜0.001 | 1.327 (1.105-1.595) | ＜0.001 |
| Cirrhosis |  |  |  |  |
| (No/Yes) | 4.303 (2.615-7.081) | ＜0.001 |  |  |
| Ascites |  |  |  |  |
| (No/Yes) | 2.439 (2.045-2.910) | ＜0.001 |  |  |
| PVTT |  |  |  |  |
| (No/Yes) | 2.778 (2.331-3.312) | ＜0.001 | 1.441 (1.187-1.751) | ＜0.001 |
| HBV-DNA(IU/ml) |  |  |  |  |
| ≤500, >500 | 1.583 (1.323-1.895) | ＜0.001 |  |  |
| Tumor multiplicity |  |  |  |  |
| (Single/Multiple) | 1.501 (1.263-1.785) | ＜0.001 |  |  |
| Tumor size |  |  |  |  |
| (＜5cm, ≥5cm) | 2.438 (2.040-2.913) | ＜0.001 | 1.595 (1.301-1.956) | ＜0.001 |
| WBC |  |  |  |  |
| (≤10/＞10*10^9/L) | 1.763 (1.128-2.757) | 0.01 |  |  |
| NLR |  |  |  |  |
| (≤2/＞2) | 1.696 (1.406-2.047) | ＜0.001 |  |  |
| RBC |  |  |  |  |
| (＜4/≥4*10^12/L) | 0.481 (0.402-0.576) | ＜0.001 |  |  |
| HGB |  |  |  |  |
| (＜110/≥110g/L) | 0.532 (0.443-0.639) | ＜0.001 |  |  |
| PLT |  |  |  |  |
| (＜100/≥100*10^9/L) | 0.410 (0.335-0.501) | ＜0.001 |  |  |
| PSL |  |  |  |  |
| (＜909/≥909) | 0.200(0.153-0.262) | ＜0.001 | 0.263 (0.183-0.380) | ＜0.001 |
| CR |  |  |  |  |
| (≤111/＞111μmoI/L) | 2.121 (1.530-2.941) | ＜0.001 |  |  |
| ALT |  |  |  |  |
| (≤50/＞50U/L) | 1.510 (1.249-1.824) | ＜0.001 |  |  |
| TBIL |  |  |  |  |
| (≤18.8/＞18.8μmol/L) | 2.025 (1.694-2.421) | ＜0.001 |  |  |
| ALB |  |  |  |  |
| (<40/≥40g/L) | 0.456 (0.367-0.567) | ＜0.001 |  |  |
| LDH |  |  |  |  |
| (≤250/＞250U/L) | 2.131 (1.705-2.664) | ＜0.001 | 1.396 (1.089-1.788) | 0.01 |
| GGT |  |  |  |  |
| (≤60/＞60U/L) | 2.270 (1.905-2.704) | ＜0.001 | 1.357 (1.095-1.681) | 0.01 |
| ALP |  |  |  |  |
| (≤125/＞125U/L) | 2.909 (2.438-3.471) | ＜0.001 | 1.403 (1.139-1.729) | ＜0.001 |
| PTA |  |  |  |  |
| (<70/≥70%) | 0.459 (0.386-0.546) | ＜0.001 |  |  |
| AFP |  |  |  |  |
| (≤400/＞400ng/ml) | 1.694 (1.420-2.021) | ＜0.001 | 1.473 (1.220-1.778) | ＜0.001 |
| CRP |  |  |  |  |
| (<5/≥5mg/L) | 1.754 (1.419-2.169) | ＜0.001 | 1.263 (1.001-1.593) | 0.05 |
